# Supplementary material for: Improved In Vitro Model for Intranasal Mucosal Drug Delivery: Primary Olfactory and Respiratory Epithelial Cells Compared with the Permanent Nasal Cell Line RPMI 2650
Source: Pharmaceutics. 2019 Aug 1;11(8):367. doi: 10.3390/pharmaceutics11080367 (PMC6723747; doi:10.3390/pharmaceutics11080367)
Supplement: Supplementary file 1 [file pharmaceutics-11-00367-s001.zip › pharmaceutics-544674-supplementary.pdf]

pharmaceutics

MDPI

Supplementary Materials: Improved In Vitro Model for Intranasal Mucosal Drug Delivery: Primary Olfactory and Respiratory Epithelial Cells Compared with the Permanent Nasal Cell Line RPMI 2650

Simone Ladel, Patrick Schlossbauer, Johannes Flamm, Harald Luksch, Boris Mizaikoff and Katharina Schindowski

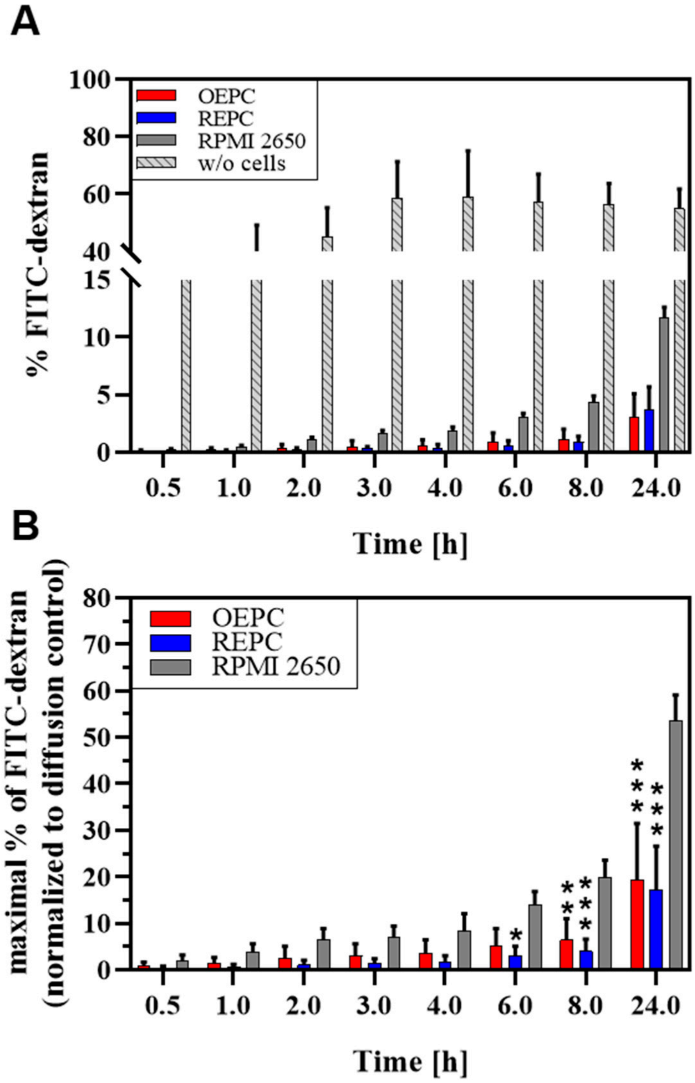

Figure S1. Permeation data referenced to control without cells (diffusion control) vs. referencing to total FITC-dextran. (**A**) Original and normalized FITC-dextran flux data. Raw data of FITC-dextran flux through OEPC (red), REPC (blue), and RPMI 2650 (grey) plotted against the time on the left y-axis and the permeation through the membrane of the transwell without cell layer (dashed bars). (**B**) As demonstrated in (**A**), an equilibration is obtained after roughly 3 h in the control insert without cells. Since this limits the maximal flux of molecules that are able to cross the cellular barrier, the normalized data are displayed here. 100% denominate the equilibrium amount of FITC-dextran that diffused to the abluminal compartment in the diffusion control (w/o cells).

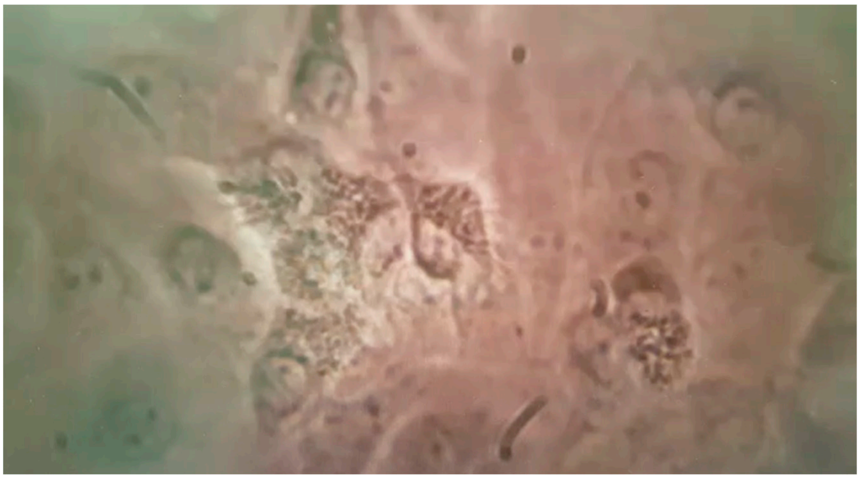

Video S1. Cilia motion in primary cell cultures of nasal olfactory epithelial cells. Ciliated cells show a beating frequency of roughly 5 Hz under submerged conditions.
